# Male bumblebees (*Bombus terrestris*) are more active and behaviourally flexible than workers

**DOI:** 10.1007/s10071-026-02061-5

**Published:** 2026-04-10

**Authors:** Pizza Ka Yee Chow, Théo Robert, Sophie Donnelly, Kevin D. Hochard

**Affiliations:** 1 Division of Psychology, University of Chester, Chester, CH14BJ UK; 2 Biosciences Institute, Newcastle University, Henry Wellcome Building, Newcastle Upon Tyne, NE17RU UK; 3 School of Biosciences, University of Sheffield, Sheffield, S10 2TN UK; 4 Neuroscience Institute, University of Sheffield, S10 2TN Sheffield, UK

**Keywords:** Pollinator, Eusocial, Insects, Colony life cycle, Insect cognition

## Abstract

Sex role differences can influence ecological and evolutionarily important traits like activity level and behavioural flexibility. In bumblebees, female workers are the main foragers for the colony, whereas males (drones) have minimal responsibility. However, males become solitary foragers once they leave the colony, suggesting that increased activity level and behavioural flexibility are crucial for their survival. Here, we conducted a laboratory experiment to compare male and female bumblebees’ active time in a novel environment (using an ‘activity’ task) as well as their colour-reward associative learning ability and behavioural flexibility (with a simultaneous two-choice discrimination-reversal colour learning task). As predicted, males were more active in the novel environment than females. Males and females showed comparable performance in learning the colour-reward association, but males demonstrated enhanced behavioural flexibility when the reward contingency changed. Males’ active time may reflect their exploratory behaviour (e.g., pre-mating patrolling), and their enhanced flexibility suggests their readiness to find new profitable flowers when exploited flowers decrease in quality. These results highlight the importance of these behavioural and cognitive traits for males, which may increase their chance of finding mates and improve their foraging efficiency.

**Supplementary Information:**

The online version contains supplementary material available at 10.1007/s10071-026-02061-5.

## Introduction

The expression of ecological and evolutionary behavioural and cognitive traits may vary within a species depending on the roles of the sexes in different fitness-related contexts (e.g., Lucon-Xiccato and Bisazza 2014; Videlier et al. 2015; Ogurtsov et al. 2018; Fuss and Witte 2019). For example, in species of bumblebee, *Bombus spp.* which show classic sex role differences (Beekman and van Stratum 1998; Duchateau 2004; Huth-Schwarz et al. 2011; Del Castillo and Fairbairn 2012; Zhao et al. 2021a), female workers who forage and care for the brood during the colony development whereas males do not forage for the colony because they do not have pollen baskets, and they are more specialised for reproduction (Thomson and Plowright 1980). In other words, males have minimal responsibility before dispersal (Goulson 2003; Baer 2003; Yadav et al. 2016; Belsky et al. 2020; Zhao et al. 2021b). When males leave their nest, they rarely return home (Goulson 2003; Kraus et al. 2009; Robert et al. 2017); they then begin solitary foraging and search for mates for reproduction. In this context, some behavioural and cognitive traits may vary between males and workers, such as activity level and behavioural flexibility, because they have fitness consequences, for instance by enhancing colony growth, facilitating the exploitation of novel food resources, or increasing mating opportunities and mating success (Reader 2015; Reader et al. 2003; Réale et al. 2007; Dukas 2013).Thus, investigating whether traits vary in expression is crucial for understanding the ultimate significance of these traits.

Active time reflects time not spent on immobile activities (e.g., resting, sleeping) (van Dixhoorn et al. 2024). Activity time has also been related to exploration (e.g., Montiglio et al. 2010; Tanaka et al. 2012), which allows individuals to discover resources and gain information about their environment (Reader 2015). More active bumblebee males can better explore their environment, increase their chances of finding an unrelated queen for reproduction and discover profitable flower patches (Kraus et al. 2009; Amin et al. 2012). Indeed, bumblebee males have been shown to fly longer and travel farther than workers (Bertsch 1984; Osborne et al. 1999; Chapman et al. 2003; Kraus et al. 2009; Wolf et al. 2012). As males leave their nest, they explore the outside world for the first time. The proxy of locomotion or overall activity level in the first exploration can be measured using adapted, established behavioural and cognitive tasks (e.g., analogue to those like the ‘open field test’, see review by (Gould et al. 2009). This type of test commonly involves placing an animal in a novel, open arena, which is typically circular, square, or rectangular in shape (Walsh and Cummins 1976). The arena may be divided into equal-sized units or marked by distances to quantify the animal’s overall locomotion and spatial utilisation. Measurements are generally taken over a predetermined time period or until the animal ceases movement (Walsh and Cummins 1976).

Behavioural flexibility, or being able to quickly adjust to environmental changes, demands and opportunities (Dukas and Bernays 2000; Chapman et al. 2003; Coppens et al. 2010a, b; Lea et al. 2020a, b), has been shown to have fitness consequences (e.g., Dukas and Bernays 2000; Nicolakakis et al. 2003). This is another trait that is expected to be particularly important for male bumblebees. When male bumblebees become solitary foragers, the cost of not being behaviourally flexible is arguably higher for them than for females who have access to resources in the colony. Behavioural flexibility can be seen, for instance, when males adjust their foraging strategy depending on the spatial structure of resources (distance between flowers, Rossi et al. 2025). This trait may increase males’ survival by allowing them to quickly respond to changes in their environment (e.g., adapting their foraging behaviour when flower quality decreases), maximise energy gain (Bertsch 1984; Brown and Brown 2020), and in turn, gain the time to search for mates. To measure behavioural flexibility, the use of the traditional discrimination-reversal learning task may be used. This task typically includes two learning phases (Shettleworth, 2010), namely, a discrimination learning phase (DLP) and a reversal learning phase (RLP). The DLP, involves presenting two stimuli to individuals, with one stimulus associated with reward whereas the other stimulus is not. Individuals have to learn this reward contingency until they meet a stringent learning criterion (typically 80% across consecutive sessions). Once this criterion is met, the RLP begins whereby the reward contingency is switched so that the previously rewarded stimulus becomes non-rewarded and the previously non-rewarded stimulus becomes rewarded. The individuals have to learn the changed reward contingency until they meet the criterion. Performance of associative learning ability in the DLP and behavioural flexibility in the RLP can be measured by the number of errors made before reaching the criterion in the respective learning phases (e.g., Tapp et al. 2003; Izquierdo and Jentsch 2012; Wascher et al. 2021).

Here, we asked whether active time and behavioural flexibility differed between male and female bumblebees. To do this, we designed an ecologically relevant experiment by creating a new environment (an ‘exploration’ task) to measure the overall activity time of male and female bumblebees on their first time leaving the nest and using the traditional two-colour discrimination-reversal learning task to assess their associative learning ability and behavioural flexibility. Previously, a few studies have compared females’ and males’ associative learning of profitable flowers’ colour and location (Dyer and Chittka 2004; Wolf and Chittka 2016; Robert et al. 2017; Frasnelli et al. 2021; Muth et al. 2021; Manning et al. 2021), and the results of these studies showed that females and males show comparable performance in associative learning. These findings allowed us to predict that males and females would show a comparable performance in associative learning in our experiment. Despite this, we were yet to explore differences in active time when bees first leave their nest and their performance in behavioural flexibility. The fact that males travel longer and farther away than females workers (Bertsch 1984; Osborne et al. 1999; Chapman et al. 2003; Kraus et al. 2009; Wolf et al. 2012) led us to predict that males would likely show a higher overall active time on their first time leaving the nest than females. Males’ flexible adjustments in foraging as solitary foragers (e.g., Rossi et al. 2025) led us to predict that males would be more behaviourally flexible than females.

## Methods

### Study species and housing

We conducted this experiment daily between May and August 2024. Bees from 5 colonies (3 colonies and 2 pocket hives purchased from commercial suppliers (BIOBEST from Biobest Belgium N.V., Westerlo, Belgium, and Koppert, UK). Two colonies were housed in a natural environment based at Toyota Manufacturing Deeside and later brought to the lab for the study when there were few individuals left in the colony. The other colony and pocket hives were housed in the lab. Bees were kept in a black box, mirroring their underground nest in the wild. The nest was attached to a transparent circular tunnel (diameter 4 cm), which had three shutters and an open end to capture the bees using a marker cage and a plunger for the experiment. The lab (room temperature 21–23^∘^c) was lit with natural daylight and artificial lights. The bees had ad libitum access to syrup and pollen during the exploration task, but reduced amounts during the learning task to increase their motivation to participate in the experiment. They had continuous access to syrup in several wax pots within the colony, and these pots were replenished once daily, after all the bees had completed the learning experiment. Bees (N = 69) were individually marked with a numbered tag (Toppa et al. 2021).

### Ethics

While there is no general animal welfare and husbandry guideline for invertebrates, this study strictly followed ASAB and ABS animal welfare guidelines and was approved by the Division of Psychology Ethics Committees, University of Chester (no: SDPC130624). Animal welfare and husbandry were carried out daily, whereby bees were fed syrup and pollen. The bees had ad libitum access to syrup and pollen before the exploration task, but we decreased the amount of syrup and pollen given to them during the learning task to increase their motivation. Enrichments (e.g., paper sticks and tissue tiles) were provided for the bees.

### Apparatus

**Fig. 1:**
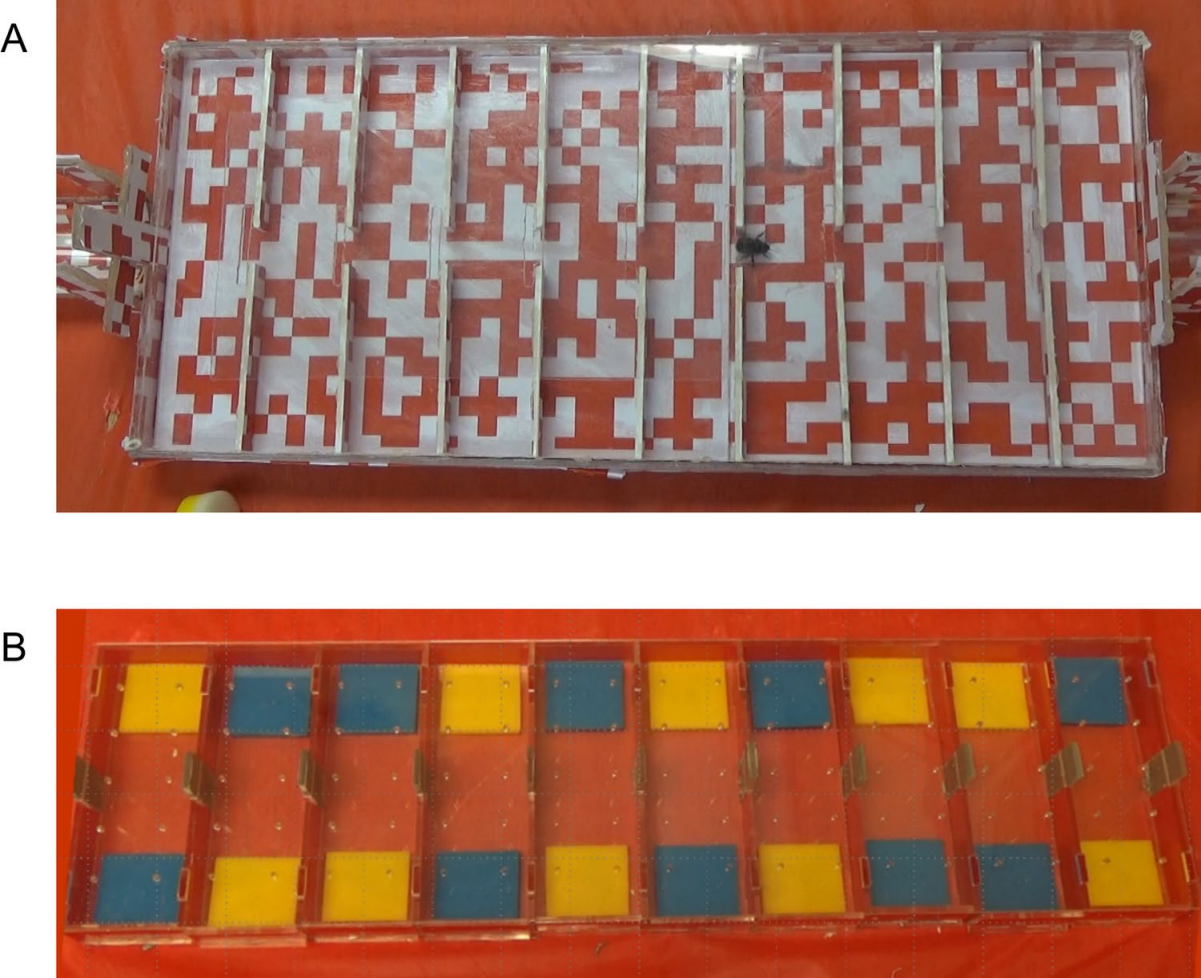
(**A**) The activity box is a novel arena that allowed individual bees to freely move between the 10 equal-sized compartments through the holes in the middle of the dividers. The floor of the box was covered with random red-and-white checkers. (**B**) The learning box that assessed individual bee’s associative learning performance in the discrimination learning phase (DLP) and behavioural flexibility in the reversal learing phase (RLP). This box also had 10 equal-sized compartments. A flower pair (blue and yellow) was presented in each compartment. A shutter was in the middle of each divider, which was lifted after the bee made a correct choice (rewarded flower)

The experiment used two apparatuses that share similar features (Fig. 1). Both apparatuses were rectangular-shaped boxes that had 10 equally divided compartments. For the exploration apparatus (Height x Width x Length: 3.3 cm × 21.8 cm × 50.9 cm, Fig. 1A), the floor of each compartment (W x H: each 4.5 × 1.2 cm) was covered with a random mosaic white-and-red square (1 cm × 1 cm) pattern. Each cell had a hole in the middle of the wall separating it from the next one, and a shutter was used to block this entrance between compartments, and at one end of the apparatus. This allowed the experimenter (PC and SD) to lift the shutter and let a bee enter/exit the experiment or go to the next compartment through the holes.

The learning apparatus was used to conduct the discrimination-reversal learning task (H x W x L: 3.1 × 15.6 × 53.6 cm, Fig. 1B). Each compartment had a pair of colour stimuli (each 4 cm × 4 cm). The colours of the stimuli were yellow (Perspex® Yellow 250) and blue (Perspex® Blue 727). These two colours are preferred by bees, and they can clearly distinguish them from each other (e.g., Raine et al. 2006; Raine and Chittka 2008, 2012; Ings et al. 2009; Strang and Sherry 2014; Evans et al. 2017). The stimuli were horizontally positioned, one on the left side and one on the right side of the compartment, equidistant from the entrance. The design of the box and the way of presenting the stimuli horizontally aimed to enhance visual information processing during the task (Rother et al. 2023). Aside from the boxes, there was a video camera, attached to a tripod, used to record the behaviour and performance of the bees. The tripod was placed around 30 cm away from the apparatus, and the video camera was 1.5 m above the box.

### Procedure

Bees went through the same standardised procedure; the start time of the experiment was standardised across colonies (typically at 8.45 am), and we tested a bee during his/her active time—that is, when a bee emerged from the colony and reached the end of the tunnel connected to the nest. A marker cage was positioned at the tunnel exit. When the bee entered the cage, it was gently closed with a plunger to collect the bee, which was then brought to the task. Each bee was tested alone. Bees first participated in the activity task, followed by the discrimination learning phase (DLP) and finally a reversal learning phase (RLP). Testing a bee was discontinued if s/he did not emerge from the colony on a test day due to a loss of motivation or death, which led to a decrease in the sample size across tasks; this procedure controlled/avoided confounding variables (e.g., fluctuation in motivation, memory decay during the learning task).

Sixty-nine bees (33 females, 36 males) left their nest for the first time to freely move in a novel environment (‘the activity box’). This task was designed to measure bees’ active time in a novel environment when they first experienced it. The task started when a bee entered his/her full body in the box and ended when the bee’s full body exited the box. During the task, no shutter was used; the bee visited any compartment as s/he wished. If a bee was inactive for 15 min in the box, the session was terminated, and the bee was brought home using the same marker cage with the plunger. A bee could reattempt a session if s/he emerged from the colony 40 min or more after the last session. The last compartment was attached with a marker cage; when the bee completed the session (i.e., left the last compartment), s/he entered the cage, which was then gently closed with a plunger. S/he was fed with 50% w/w sucrose ad libitum (a food source preferred by both sexes, and they showed a preference for higher concentrations over lower ones) (Bailes et al. 2018; Pamminger et al. 2019; Brown and Brown 2020); this aimed to assess whether they were responsive to sucrose (i.e., extends its proboscis toward the sucrose), and the amount drunk was measured.

The bees that emerged from the colony after the activity task had an additional ‘training phase’. This training phase included two sessions that served two purposes: 1) to allow the bees to familiarise themselves with the procedure of the learning task, and 2) to ensure the bees had high motivation for the learning task. In both training sessions, a shutter was added to block the hole between compartments so that the bees had to explore both the right and left sides of the box. The shutter between the compartments was lifted every 30 s, and the bees had to go through all 10 compartments to be considered to have passed the training phase. After each training session, the bees were rewarded with 50% w/w sucrose ad libitum (Bailes et al. 2018; Pamminger et al. 2019; Brown and Brown 2020) when s/he left the last compartment. The box was thoroughly cleaned using water and alcohol wipes before the next bee was trained, with the following steps: 1) we used tissue papers to absorb bees’ dejections; 2) we used alcohol wipes (70% ethanol) to clean each compartment and all sides of the box; 3) we used hot water to rinse the compartments; and finally, 4) we used tissue papers to dry all compartments and reset a session for the next bee. These cleaning steps are aimed at removing any scent left from the previous bee. After cleaning, the box was reset for the next bee.

The bees that completed the training phase were tested in the learning task on the following day to avoid overfeeding, with full completion of the training serving as an indicator of motivation. The learning task was used to measure bees’ colour-reward associative learning ability in the DLP and behavioural flexibility in the RLP. A bee participated in 1–2 sessions daily (as a motivation measure and to avoid over-feeding) until they had completed the learning phases (see learning criteria below), did not return to the task or died (participation days ranged from 1 to 24 days). Each session included 10 flower pairs (i.e., 1 pair per compartment, bees made 10 choices in total per session). The flower pair was blue and yellow. Each bee was randomly assigned to associate one flower colour with a reward (0.01 ml 50% w/w sucrose drop) and the other flower colour with a control (0.01 ml water drop). The drop on each flower was placed at its centre. The size of the drops was < 1 mm in diameter, which required the bees to walk close to the stimuli and use their antenna to detect whether the drop was water or sucrose. The presentation of the flower pairs was pseudo-randomised within and across sessions. Within sessions, the same flower colour was presented on the same side for no more than two consecutive compartments to avoid bees developing side bias. The same flower colour was shown on the left side five times (out of 10) and another five times on the right side. Across sessions, the same colour pair was not presented in the same compartment more than two consecutive times. All the bees experienced the same colour sequence to control for the order effect on the experience.

Bees could explore both flowers, and their first choice (indicated by at least half of his/her body on a flower) was marked as either ‘correct’ (reward) or ‘incorrect/error’ (control). When a bee chose the rewarded flower, the shutter in the middle of the divider was lifted, and the bee passed to the next compartment. When the bee made an error, s/he had to visit the other flower (i.e., correctional choice) before going to the next compartment. Choices were recorded both by direct observation and by the video camera. The criterion for completing a learning phase was that a bee had participated in the task daily and that the bee made ≥ 8 out of 10 rewarded first choices (i.e., ≥ 80% correct) in two consecutive sessions. The bee was brought back home when s/he completed the task and went to the second session if s/he emerged from the colony 40 min or more after the last session. After each session, the cleaning steps outlined in the activity task above were applied to the box, the compartments, and the flowers before resetting them for the next bee.

Bees went to the RLP the day after they had completed the DLP. The RLP had the same protocol and learning criterion as the DLP. In the RLP, the bees had to unlearn the previous colour-reward association (e.g., B + Y-), and relearn that the previously unrewarded colour became rewarded (e.g., B-Y +) until they reached the same learning criterion set in the DLP (80% choices on rewarded colour).

### Behavioural measurement

Active time in the compartments when the bee first experienced the novel apparatus was analysed (to better reflect the behaviour of a bee leaving the nest the first time). Active time in the compartments was measured in seconds, and the recording started when a bee’s full body entered the apparatus (the first compartment) until its full body left the apparatus (the last compartment). From these recordings, we obtained the active time of each bee in each compartment, total active time across compartments (i.e., the sum of active time in each compartment), the frequency of visiting each compartment, and mean active time in each compartments (i.e., total active time in each compartment divided by the number of visits to that compartment).

The associative learning and behavioural flexibility performances were the number of errors made before reaching the learning criterion in the DLP and RLP, respectively (e.g., Tapp et al. 2003; Izquierdo and Jentsch 2012; Wascher et al. 2021). We also adjusted the learning criterion to include bees that had not returned to the task but showed significant learning, when a bee had 80% of correct responses in a single session with at least 5 consecutive correct responses or more in that session.

We additionally measured the size of each bee, which was estimated using the inter-tegular span (ITS) (Cane 1987; Hagen and Dupont 2013). We used a digital calliper to obtain the distance (mm) between the tegulae. Sucrose consumption was measured as the amount (ml) consumed after the activity task, using a syringe with a 0.02 ml graduation.

### Statistical analysis

All analyses were conducted using R (version 4.4.1), and the significance level was set as two-tailed *p* ≤ 0.05. Data were analysed using Generalised Linear Models and Generalised Linear Mixed Models (GLMM) from the ‘glmmTMB’ package (Magnusson et al. 2017), pairwise contrasts with Tukey correction from the ‘emmeans’ package (Lenth and Piaskowski 2026), a binomial test and individual analysis (Sokal and Rohlf 1995). All GLMM models included bee ID as a random variable to maximise model convergence. Model fits were checked with the package ‘DHARMa’ (Hartig 2025). Multicollinearity was checked after running each model using the Variance Inflation Factor (VIF) (< 5) and tolerance (0.25). These packages generated estimates, standard errors, z and p values for the results.

*Activity task.* A GLMM with gamma log link distribution was used to model the positively skewed, non-negative but continuous variables (e.g., active time) (Ng and Cribbie 2017). Main analyses included between-group level to test whether: 1) sex predicted the total active time in all the compartments (Table 1a); 2) sex, compartment number, and their interaction influenced the total active time in each compartment (Table 1b); 3) sex, compartment number, and their interaction influenced the mean active time in each compartment (Table 1c). Additional analyses for the activity task included: 1) a GLMM gamma log link distribution test on sex difference in body size (Table 1e); 2) a Poisson log link distribution test to examine the effects of sex, compartment number and their interaction on the frequency of visits to each compartment, (Table 1d), with post hoc analyses using pairwise contrasts with Tukey corrections to compare sex differences in frequency of visits to each compartment (Table S1); 3) a GLMM gamma log link distribution test to analyse sex differences in drinking amount after the activity task (Table 1f); 4) within-sex analyses using a GLMM to examine the effects of body size (ITS) and the total active time in all compartments on sucrose consumption (Table 1g-h).

**Table 1:** GLMM results of the activity task. Models a–f use female as the reference group

	Response variable	Fixed variable(s)	Est	S.E	*z*	*p*
a	Total active time	Sex (Male)	1.13	0.30	3.77	** < 0.001**
b	Active time in each compartment	Sex (Male)	0.90	0.30	3.03	**0.002**
		Compartment number	-0.20	0.02	-10.14	** < 0.001**
		Sex (Male)* compartment number	< 0.01	0.03	0.13	0.899
c	Mean active time in each compartment	Sex (Male)	0.26	0.19	1.37	0.171
		Compartment number	-0.12	0.02	-7.05	** < 0.001**
		Sex (Male)* compartment number	0.04	0.02	1.63	0.103
d	Frequency in each compartment	Sex (Male)	0.80	0.20	4.00	** < 0.001**
		Compartment number	-0.10	0.01	-8.17	** < 0.001**
		Sex (Male)* compartment number	-0.05	0.01	0.01	** < 0.001**
**Between-sex analysis**						
e	Inter-tegular span (ITS) (mm)	Sex (Male)	0.04	0.04	1.00	0.317
f	Amount drank (ml)	Sex (Male)	-0.06	0.25	-0.22	0.828
	Within-sex analysis					
g	Males: Amount drank (ml)	Inter-tegular span (ITS) (mm)	0.60	0.31	1.94	0.053
		Active time	0.01	0.01	1.37	0.170
h	Females: Amount drank (ml)	Inter-tegular span (ITS) (mm)	0.93	0.50	1.86	0.063
		Active time	-0.31	0.09	-3.49	** < 0.001**

This table shows fixed variables, estimates (est), standard error (S.E), *z* and *p* values. Bold values indicate significance, *p* < 0.05

*Discrimination-reversal learning task.* For each learning phase, two models were run to examine learning performance using GLMM Poisson log link distribution. In the first model, we only included bees that had met the learning criterion (80% correct responses for two consecutive sessions). This model included a fixed factor, sex, and the response variable was the number of errors made before reaching the learning criterion (Table 2c). We then included bees that had met the adjusted learning criterion (i.e., met the 80% learning criterion with 5 or more consecutive correct responses in a session) and reran the model (Table 2d).

**Table 2:** GLMM results on DLP performance

	Response variable		Fixed variable(s)	Est	S.E	*z*	*p*
a	Number of errors (with adjusted criterion)	Initial choice (very first choice) in DLP		0.71	0.87	0.82	0.413
b	Number of errors (with adjusted criterion)	DLP Assigned rewarded colour (blue or yellow)		0.82	0.92	0.89	0.372
c	Number of errors	Sex (Male)		-1.79	0.78	-2.30	**0.022**
d	Number of errors with adjusted criterion	Sex (Male)		-0.98	0.66	-1.48	0.139
e	Completion of DLP	Inter-tegular span (ITS) (mm)		1.29	1.10	1.17	0.241
		Active time		0.16	0.21	0.73	0.468
		Amount drank (ml)		23.43	11.63	2.01	**0.044**
Within-sex analysis							
f	Males (*n* = 10): Number of errors (with adjusted criterion)		Inter-tegular span (ITS) (mm)	-0.54	0.52	-1.04	0.298
			Amount drank (ml)	4.15	2.36	1.76	0.079
			Active time	-0.05	0.07	-0.70	0.482
g	Females (*n* = 9): Number of errors (with adjusted criterion)		Inter-tegular span (ITS) (mm)	0.88	0.86	1.03	0.304
			Amount drank (ml)	1.87	1.34	1.39	0.163
			Active time	-0.61	0.26	-2.32	**0.021**

Model c includes the number of errors when bees met the stringent learning criterion (80% correct responses for two consecutive learning sessions). Model d includes the number of errors when bees met the adjusted learning criterion (80% correct responses in a session with 5 or more consecutive correct responses in the session). Within-sex analyses of female (Model g) include 9 females, as two females lost their tags before measuring body size, and thus reduced the sample size for females

Models a-e are group-level analysis, and Models f and g are within-sex analysis. Models c–d use female as the reference group. This table shows fixed variables, estimates (est), standard error (S.E), *z* and *p* values. Bold values indicate significance, *p* < 0.05

*Between-group analyses of each learning phase.* In the DLP, we conducted between-group analyses using pairwise contrasts with Tukey corrections for multiple comparisons to examine whether females and males who had and had not completed the learning phase differed in body size (inter-tegular span) (Table S2a), sucrose consumption (Table S2b), and total active time in the activity task (Table S2c). For the RLP, all bees met the (adjusted) learning criterion (i.e., completed the task), and thus, between-group analyses reflected sex differences. We ran GLMM with gamma log link distribution to examine body size in one model (Table 3c), sucrose consumption in the activity task (Table 3d) in another model, and total active time in all compartments in the final model (Table 3e).

**Table 3:** GLMM results of RLP performance

	Response variable	Fixed variable(s)		Est	S.E	*z*	*p*
a	Number of errors (the adjusted learning criterion)		Learning session (last DLP session)	-0.98	0.10	-9.45	** < 0.001**
b	Number of errors		Learning phase (RLP)	0.96	0.12	7.83	** < 0.001**
c	Inter-tegular span (ITS) (mm)		Sex (Male)	0.04	0.07	0.54	0.592
d	Amount drank (ml)		Sex (Male)	-0.93	0.81	-1.15	0.251
e	Total active time (secs)		Sex (Male)	0.43	0.38	1.11	0.266
Within-sex analysis							
f	Males (*n* = 7): Number of errors (with adjusted criterion)		Inter-tegular span (ITS)	-0.25	0.14	-1.72	0.085
			Amount drank (ml)	-2.59	1.54	-1.68	0.093
			Active time	0.02	0.02	0.96	0.335
g	Females (*n* = 7): Number of errors (with adjusted criterion)		Inter-tegular span (ITS)	1.32	0.32	4.17	** < 0.001**
			Amount drank (ml)	0.37	0.47	0.79	0.430
			Active time	-0.07	0.12	-0.53	0.596

Model a uses the first RLP session as the reference group, and Model b uses the DLP session as the reference group; both include bees (n = 15) that met the adjusted learning criterion (≥ 80% correct responses in a session with at least five consecutive correct responses). Models c–e are between-group comparisons for the 8 females and 7 males who completed the RLP. One female lost her tag before body size measurement, reducing the female sample size. Females served as the reference group in these models

This table shows fixed variables, estimates (est), standard error (S.E), *z* and *p* values. Bold values indicate significance, *p* < 0.05

*Within-sex analyses of each learning phase.* For bees that had completed each learning phase, we carried out within-sex analyses to examine predictors for learning performance. We used GLMM Poisson log link distribution to examine whether their body size (inter-tegular span), sucrose consumption in the activity task, and total active time in all compartments predicted their DLP learning performance in one model (Table 2f-g) and RLP performance (behavioural flexibility) in another model (Table 3f-g).

*Additional analysis.* For bees that had met the unadjusted DLP learning criterion (n = 15), we further used GLMM Poisson log link distribution to examine whether their DLP performance was affected by their initial choice (Y/B) in one model (Table 2a), and their assigned initial reward colour (Y/B) in another model (Table 2b). A binomial link distribution was used to examine whether inter-tegular size (ITS), active time in the activity task, and sucrose consumption would predict whether a bee completed the DLP or not (Table 2e).

*Performance differences between learning phases.* A GLMM Poisson log link distribution was used to compare the number of errors in the last session of the DLP and the first session of the RLP (Table 3a). A second GLMM compared the total number of errors made before reaching the learning criterion in the DLP and the RLP (Table 3b). A binomial test was used to examine whether bees’ first choice on the previously rewarded colour was significantly above chance. Individual analyses were also conducted to understand whether bees had learned the DLP colour-reward association.

## Results

### Sex and activity levels in a new environment

**Fig. 2:**
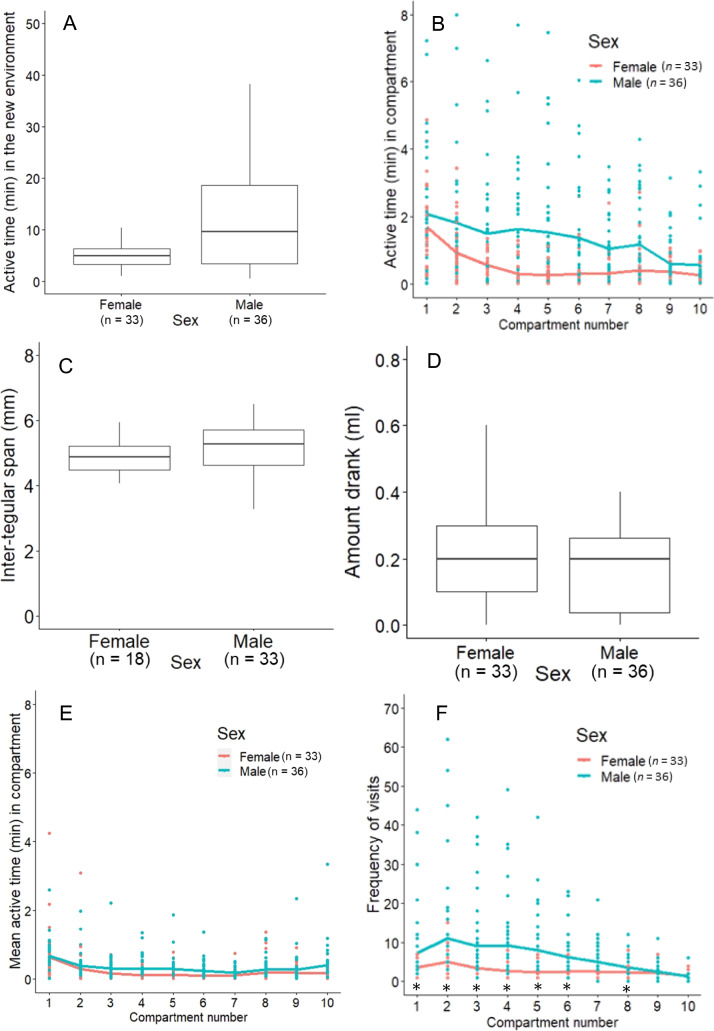
The activity task.** (A)** Distribution of the total active time (mins) in all compartments for females and males. **(B)** Individual total active time (dots) and the mean of total active time (mins) in each compartment (line) for females (red) and males (blue). **(C)** ITS (mm) of females and males. **(D)** Sucrose consumption (ml) of females and males. **(E)** Individual means (dots) and the mean active times (mins) of all individuals (line) per visit in each compartment for females and males. **(F)** Individual total visits (dots) and mean (lines) frequencies of visits across individuals to each compartment for females and males. * indicates *p* < 0.05

The total active time was significantly longer and showed a wider variation in males (median = 11 min) than in females (median = 5 min) (GLMM: *p* < 0.001, Fig. 2A, Table 1a). Active time per compartment was also significantly higher in males than in females (*p* = 0.002, Fig. 2B, Table 1b). Bees’ active time significantly decreased over successive compartments (*p* < 0.001), but no interaction effect was detected between sex and compartment number (*p* = 0.899, Table 1c).

Males’ higher active time in each compartment may be driven by longer but fewer visits, equally frequent visits, or shorter but more frequent visits in each compartment. We further analysed males’ movement strategy by examining whether males and females spent different amounts of time per visit in each compartment. We divided the time spent in each compartment by the number of visits to that compartment to obtain the mean time spent per visit. The between-group analyses showed that bees significantly decreased their mean active time in successive compartments (*p* < 0.001, Table 1c). Males and females had comparable mean active time in each compartment (*p* = 0.171). The interaction of sex and compartment number on mean active time was not significant (*p* = 0.103, Fig. 2E). Frequency of visits significantly decreased over successive compartments (*p* < 0.001); bees visited compartments closer to the exit less frequently than those near the entrance. Males visited each compartment more often than females (*p* < 0.001, Fig. 2F, Table 1d), and the interaction effect between sex and compartment number significantly affected the frequency of visits (*p* < 0.001). Pairwise contrasts of sex differences in the frequency of visits to each compartment further showed that males visited the compartments close to the entrance and in the middle of the box more often (Fig. 2F, Table S1). Males and females who participated in this task had a comparable body size (ITS) (*p* = 0.317, Fig. 2C, Table 1e) and sucrose consumption (*p* = 0.828, Fig. 2D, Table 1f). The additional within-sex analyses showed that males’ sucrose consumption was not associated with body size or total active time (Table 1g), whereas females’ sucrose consumption was associated with less active time (*p* < 0.001, Table 1h).

### Sex and performance in learning a colour-reward association

**Fig. 3:**
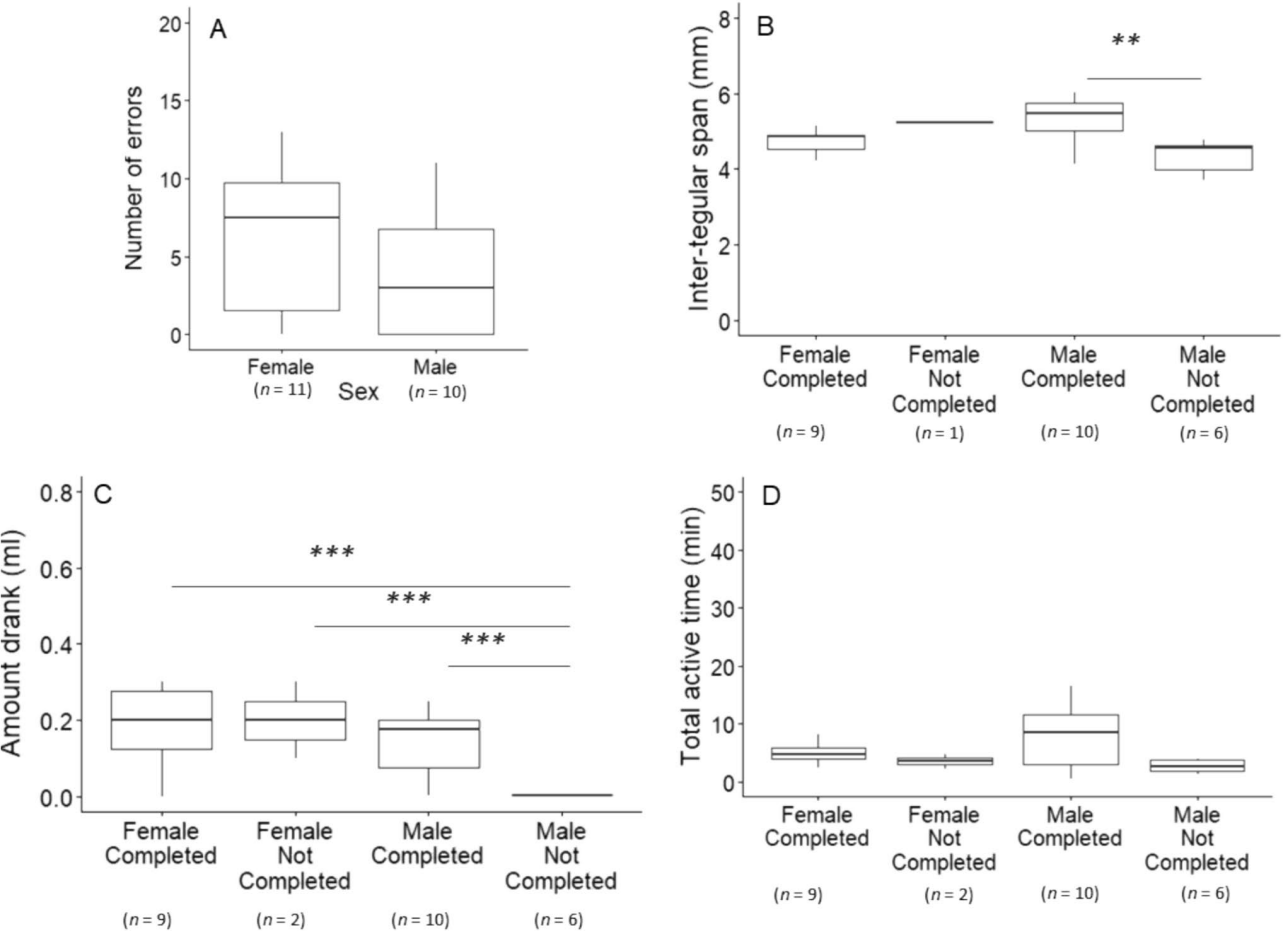
Discrimination learning phase (DLP). **(A)** The number of errors before reaching the learning criterion for both sexes (including bees with the adjusted criterion). **(B-D)** Between-group comparisons included females who completed the DLP, females who did not complete the DLP, males who completed the DLP, and males who did not complete the DLP. The ‘completed’ group included bees with the adjusted criterion, whereas the ‘not-completed’ group included bees who participated in DLP but did not reach the criterion. Note that the sample size varies by analysis, as a few bees lost their tags before body size measurements. Comparisons of the **(B)** ITS (mm), **(C)** sucrose consumption (ml), and **(D)** total active time (min) in the activity task. ** *p* < 0.01, *** < 0.001

Of the 69 bees, 29 bees (13 females, 16 males) emerged and participated in the DLP the next day after the activity task. 14 bees did not return after a few sessions (median = 4), and fifteen bees (8 females, 7 males) met the DLP learning criterion in 1 to 10 sessions (median = 4). Of these 15 bees, 7 bees first chose blue, and 8 first chose yellow, which may reflect their innate colour preference (Gumbert 2000). However, the number of errors made was neither affected by the bees’ first choice (*p* = 0.413, Table 2a) nor the initial assigned reward colour (*p* = 0.372, Table 2b). Males made significantly fewer errors than females (*p* = 0.022, Table 2c) but this significance disappeared after including a few more bees that had stopped returning to the task but showed significant learning (adjusted criterion: a single session with 80% correct and ≥ 5 consecutive correct choices, *p* ≤ 0.031) (*n* = 21, *n*_Female_ = 11, *n*_Male_ = 10, *p* = 0.139) (Fig. 3A, Table 2d).

For the 29 bees that had participated in DLP, higher sucrose consumption (in the activity task) increased the likelihood of successful completion (amount drank: p = 0.044, Table 2e). We further explored whether body size, sucrose consumption and total active time in the activity task differed across males and females who did or did not complete the DLP (Table S2). Between-group pairwise comparisons with Tukey corrections for multiple comparisons showed that the male completed group had a significantly larger body size than the male not-completed group (ITS: *p* = 0.008, Fig. 3B, Table S2a). Notably, the male not-completed group significantly consumed less sucrose than the other three groups (Fig. 3C, Table S2b). The four groups did not differ significantly in total active time in the activity task (Fig. 3D, Table S2c). The bees that had completed the learning phase allowed us to conduct analyses regarding the possible predictors for the DLP performance within each sex. Within-male analysis showed that none of the factors predicted males’ DLP performance (*n* = 10, Table 2f), whereas within-females analyses showed that females with a higher total active time made fewer errors (active time: *p* = 0.021, Table 2g).

### Sex and behavioural flexibility

**Fig. 4:**
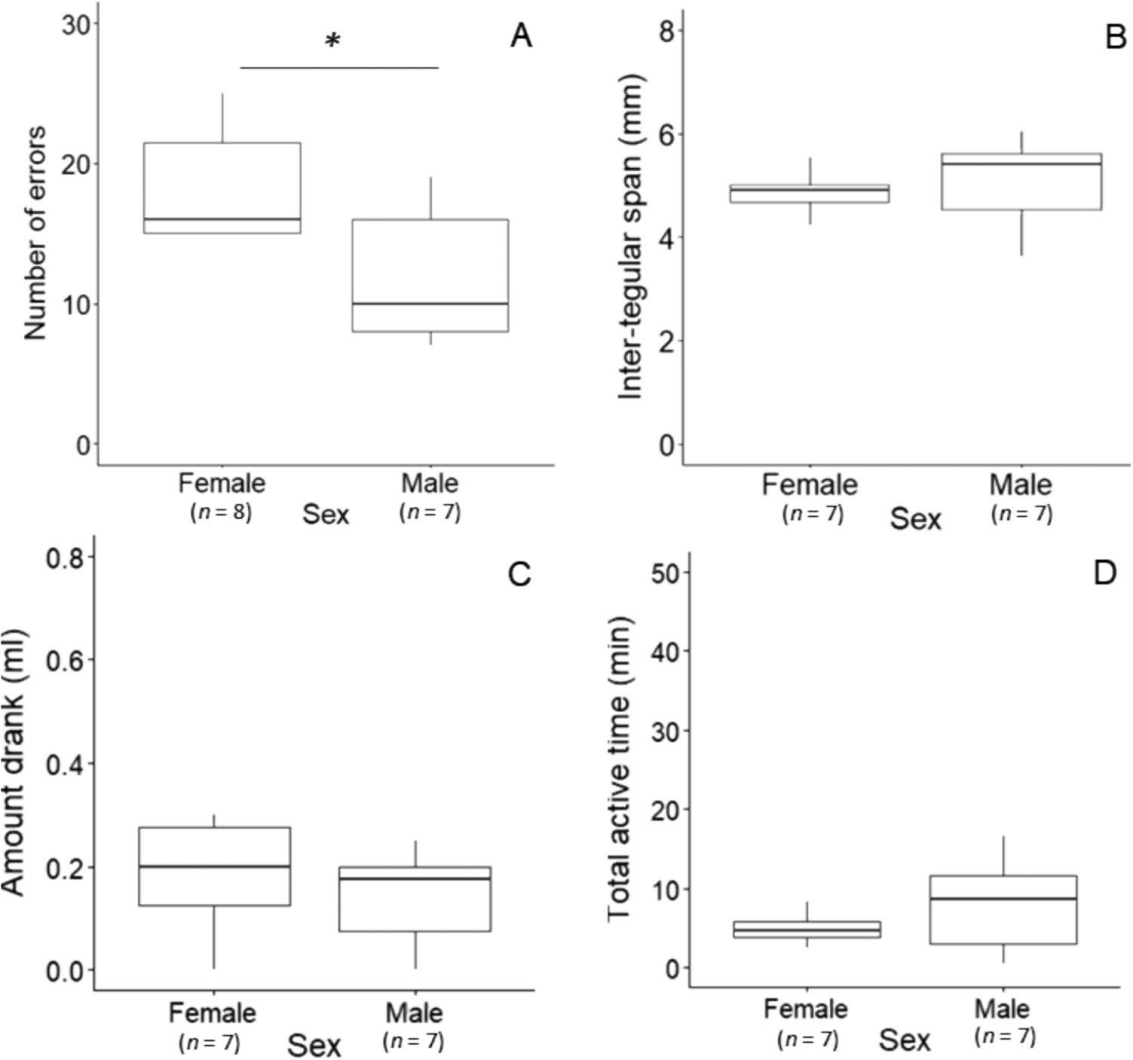
Reversal learning phase (RLP). All bees met the adjusted learning criterion, and the between-group comparisons reflected sex comparisons in (**A**) the number of errors (behavioural flexibility), (**B**) ITS (mm), (**C**) amount drank (ml) and (**D**) total active time (min). Note that one female lost her tag after completing the RLP, and thus led to a lower sample size for the female group in B-D analysis. *p < 0.05

Fifteen bees (8 females, 7 males) that had met the DLP (unadjusted) learning criterion participated in the RLP on the next day. Thirteen bees (8 females, 5 males) met the (unadjusted) RLP criterion, and all bees (8 females and 7 males) met the adjusted RLP criterion. In the first RLP session, most females (88%) and males (86%) first chose the previously rewarded colour significantly above chance (binomial exact *p* = 0.003, *n* = 15). Both females and males made significantly more errors than random on the first five choices (pooled consecutive binomial tests: females: *χ*^2^_16_ = 41.59, p < 0.001; Males: *χ*^2^_14_ = 30.50, p < 0.01, Note S1). Bees also made significantly more errors in the first RLP session than in their last DLP session (*p* < 0.001, Table 3a). Overall, bees made significantly more errors before reaching the criterion in the RLP than in the DLP (*p* < 0.001, Table 3b), showing that the RLP was more difficult and suggesting that bees had learned the DLP colour-reward association and were not relying on any hypothetical cues from the reward itself. In the RLP, males made significantly fewer errors than females (*p* = 0.023, *n* = 15, Fig. 4A). These results are retained if we only included bees that met the unadjusted RLP criterion (*p* = 0.013, *n* = 13) or excluded a female who made extensive errors before reaching the criterion (*p* = 0.049, *n* = 14).

All bees completed the RPL, and the between-group comparisons reflected sex comparisons. Additional between group analyses showed that no significant differences were detected between sexes in body size (ITS: *p* = 0.592, Fig. 4B, Table 3c), sucrose consumption (*p* = 0.251, Fig. 4C, Table 3d) or total active time during the activity task (*p* = 0.266, Fig. 4D, Table 3e). Additional within-sex analyses showed that none of these factors predicted males’ RLP performance (ITS: *p* = 0.085; amount drank: *p* = 0.093; active time: *p* = 0.335, Table 3f) whereas females who had a larger body size made more errors (ITS: *p* < 0.001; amount drank: *p* = 0.430; active time: *p* = 0.596, Table 3g).

## Discussion

Animal species with strong sex roles may exhibit different behavioural and cognitive traits that hold ecological and evolutionary significance (e.g., Lucon-Xiccato and Bisazza 2014; Videlier et al. 2015; Ogurtsov et al. 2018; Fuss and Witte 2019). By using a bumblebee species that shows clear sex role differences, we examined whether female workers and male drones differed in active time and behavioural flexibility. Key findings included that bumblebee males were more active as they showed a higher active time in a new environment the first time, bumblebee males showed comparable colour associative learning ability to females, and males were more behaviourally flexible as they made fewer errors in the RLP than females.

The fact that male bumblebes show a higher active time than females could not be attributed to body size or sucrose consumption, but may be related to the fact that drones generally fly further (disperse over greater distances) than workers during their dispersal (Bertsch 1984; Osborne et al. 1999; Chapman et al. 2003; Kraus et al. 2009; Wolf et al. 2012), suggesting a high time spent in flight when exiting their colony. In addition, we did not determine the age of the individual bees, and the higher activity time observed in males may potentially be related to age differences between males and females. Indeed, age has been shown to be negatively related to activity time and flight distance in female bumblebees (Gilgenreiner and Kurze 2024) and it is unknown if this is also true for males. Therefore, we suggest that future studies measure the age of the individual bees to factor its possible effect on males and workers activity levels. However, the active time in a novel environment the first time may be related to other traits, such as exploration tendency or anxiety-related traits (Montiglio et al. 2010; Tanaka et al. 2012). Considering the frequency of entering each compartment, males visited most compartments more often than females in their first experience, possibly suggesting exploration. This possibility is strengthened by the result that males made equally long but more frequent visits to each compartment than females. Relating these results to ecological contexts, bumblebee males generally disperse in a random direction from their nest, and travel far away from it (Osborne et al. 1999; Chapman et al. 2003; Kraus et al. 2009; Wolf et al. 2012). They patrol after dispersal; males create a flight path, marking different places with their scent to attract potential mates, and regularly patrol these places (Alcock et al. 1978; Valterová et al. 2019). These behaviours could allow males to explore their environment more than workers, and increase their chances of discovering profitable flowers after their dispersal (Kraus et al. 2009), as well as potentially increasing their likelihood of finding an unrelated queen for reproduction (Amin et al. 2012).

In the DLP, males showed comparable associative learning performance to females, which is consistent with previous studies that have used different setups (e.g., stimuli presented in an array or two-choice task) and conditions (e.g., natural or laboratory) (Wolf and Chittka 2016; Muth et al. 2021). When examining the factors that affected DLP performance, within-female analysis showed that active time was positively correlated with DLP performance. As discussed above, active time may reflect exploration tendency; a previous study has shown that such a tendency is positively correlated with associative learning ability, which can benefit the colony when workers efficiently exploit newly emerged food sources (Raine and Chittka 2008). In contrast, within-male analysis showed no relationship between associative learning and active time or other tested factors (i.e., body size, sucrose consumption). Despite this, the between-group analyses revealed that males who completed the DLP had a larger body size and drank more sucrose than the males who never completed the DLP. These results suggest that a larger body size may bring an advantage for males in learning (Del Castillo and Fairbairn 2012). It is possible that a larger body size may increase sucrose consumption; however, further investigations (e.g., increased energy expenditure in the activity task, sucrose sensitivity, or responsiveness) are encouraged to clarify the potential link between active time (activity) and associative learning abilities.

In the RLP, most bumblebees’ first choice was on the previously rewarded colour in the first session. Bees also made more errors before reaching the criterion of the RLP than in the DLP. These results suggest that bees perseverated on the previously rewarded stimulus (Strang and Sherry 2014), or that the previously rewarded stimulus interfered with learning by acting as a ‘distractor’ when bees had to relearn the reward contingency (Dyer and Chittka 2004). As predicted, males were more behaviourally flexible (i.e., made fewer errors) than females, which could not be attributed to differences in body size, sucrose consumption or active time in the activity task. Within-female analysis showed that body size was a predictor of their RLP performance. Workers’ size was negatively correlated with RLP performance, which may be explained by large and small workers differing in their foraging roles; larger bees invest more in learning highly rewarding flowers (like in our experiment) while small bees were more readily accepting flowers of all qualities (Frasnelli et al. 2021).Thus, large workers may have formed a stronger association between colour and reward during the DLP than smaller workers, which could have made it difficult for them to relearn the colour-reward contingency in the RLP. Unlike females, none of the tested factors could predict males’ RLP performance. Despite this, the males who participated in RLP were those who had completed the DLP and were larger in body size than their male counterparts who never completed the DLP, suggesting a possible link between body size and behavioural flexibility. Nevertheless, males’ overall enhanced flexibility may reflect their readiness to respond to changes in their environment, which could be ecologically adaptive. For example, adjusting their foraging decisions in response to a change in flower quality could help them to maximise energy gain, and the time spent searching for mates. Arguably, the costs of not being flexible are higher for males than for females as females can continue to access resources in the colony. Females’ lower behavioural flexibility could also be linked to their flower constancy, an adaptive trait for the workers (e.g., Chittka et al. 1999). This would thus suggest that males have lower to no flower constancy. However, it would require additional specific experiments to confirm this hypothesis. It should also be noted that a previous study has shown an upside for some workers to make more ‘mistakes’ (i.e. be less flower constant) as it can lead them to discover and exploit new profitable food sources and collect more resources for the colony (Evans et al. 2017). Therefore, future studies could conduct a second (or more) reversal to further assess the learning strategy of males, and whether sexes differ in relearning the colour-reward association across several reversals.

In conclusion, we provided evidence that a longer active time when males first leave their nest, and behavioural flexibility are two key behavioural and cognitive traits for male bumblebees in the context of their dispersal. Drones outperforming workers in their activity and behavioural flexibility could be related to the pressure of being solitary foragers. Our results call for broader assessments of additional ecologically and evolutionarily important traits that may vary between sexes with different roles, particularly given that males’ cognition has often been overlooked (e.g., Belsky et al. 2020; Muth et al. 2021). Overall, these results illustrate how sex role differences may shape sex-specific behaviour and cognition, as it has been shown in other animal species (e.g., Lucon-Xiccato 2022).

## Supplementary Information

Below is the link to the electronic supplementary material.

Supplementary file 1.

## Data Availability

Metadata and r code are freely accessible on OSF (https://osf.io/3j75g/?view_only=9d7891976466476d8c721e4a4812bd13)

## Declarations

### Pre-print

An earlier version of this study has been published in BioRxiv: doi: 10.1101/2025.03.28.645856.

## References

[CR1] Alcock J, Barrows EM, Gordh G et al (1978) The ecology and evolution of male reproductive behaviour in the bees and wasps. Zool J Linn Soc 64:293–326

[CR2] Amin MR, Bussière LF, Goulson D (2012) Effects of male age and size on mating success in the bumblebee *Bombus terrestris*. J Insect Behav 25:362–374

[CR3] Baer B (2003) Bumblebees as model organisms to study male sexual selection in social insects. Behav Ecol Sociobiol 54:521–533

[CR4] Bailes EJ, Pattrick JG, Glover BJ (2018) An analysis of the energetic reward offered by field bean (*Vicia faba*) flowers: nectar, pollen, and operative force. Ecol Evol 8:3161–3171 29607015 10.1002/ece3.3851 PMC5869266

[CR5] Beekman M, van Stratum P (1998) Bumblebee sex ratios: why do bumblebees produce so many males? Proc R Soc Lond B Biol Sci 265:1535–1543

[CR6] Belsky JE, Camp AA, Lehmann DM (2020). The importance of males to bumble bee (Bombus Species) nest development and colony viability. Insects 11.: 10.3390/insects11080506 10.3390/insects11080506 PMC7469185 32764336

[CR7] Bertsch A (1984) Foraging in male bumblebees (*Bombus lucorum* L.): maximizing energy or minimizing water load? Oecologia 62:325–336 28310885 10.1007/BF00384264

[CR8] Brown M, Brown MJF (2020) Nectar preferences in male bumblebees. Insectes Soc 67:221–228

[CR9] Cane JH (1987) Estimation of bee size using intertegular span (Apoidea). J Kans Entomol Soc 60:145–147

[CR10] Chapman RE, Wang J, Bourke AFG (2003) Genetic analysis of spatial foraging patterns and resource sharing in bumble bee pollinators. Mol Ecol 12:2801–2808 12969482 10.1046/j.1365-294x.2003.01957.x

[CR11] Chittka L, Thomson JD, Waser NM (1999) Flower constancy, insect psychology, and plant evolution. Naturwissenschaften 86:361–377

[CR12] Coppens CM, de Boer SF, Koolhaas JM (2010a) Coping styles and behavioural flexibility: towards underlying mechanisms. Philos Trans R Soc Lond B Biol Sci 365:4021–4028 21078654 10.1098/rstb.2010.0217 PMC2992750

[CR13] Coppens CM, de Boer SF, Koolhaas JM (2010b) Coping styles and behavioural flexibility: towards underlying mechanisms. Philos Trans R Soc Lond B Biol Sci 365:4021–4028. 10.1098/rstb.2010.0217 21078654 10.1098/rstb.2010.0217 PMC2992750

[CR14] Del Castillo RC, Fairbairn DJ (2012) Macroevolutionary patterns of bumblebee body size: detecting the interplay between natural and sexual selection. Ecol Evol 2:46–57 22408725 10.1002/ece3.65 PMC3297177

[CR15] Duchateau MJ (2004) Sex ratio variation in the bumblebee *Bombus terrestris*. Behav Ecol 15:71–82

[CR16] Dukas R (2013) Effects of learning on evolution: robustness, innovation and speciation. Anim Behav 85:1023–1030

[CR17] Dukas R, Bernays EA (2000) Learning improves growth rate in grasshoppers. Proc Natl Acad Sci U S A 97:2637–2640 10706621 10.1073/pnas.050461497 PMC15981

[CR18] Dyer AG, Chittka L (2004) Fine colour discrimination requires differential conditioning in bumblebees. Naturwissenschaften 91:224–227 15146269 10.1007/s00114-004-0508-x

[CR19] Evans LJ, Smith KE, Raine NE (2017) Fast learning in free-foraging bumble bees is negatively correlated with lifetime resource collection. Sci Rep 7:496 28356567 10.1038/s41598-017-00389-0 PMC5428240

[CR20] Frasnelli E, Robert T, Chow PKY et al (2021) Small and large bumblebees invest differently when learning about flowers. Curr Biol 31:1058-1064.e3 33373638 10.1016/j.cub.2020.11.062

[CR21] Fuss T, Witte K (2019) Sex differences in color discrimination and serial reversal learning in mollies and guppies. Curr Zool 65:323–332 31263491 10.1093/cz/zoz029 PMC6595423

[CR22] Gould TD, Dao DT, Kovacsics CE (2009) The Open Field Test. Mood and Anxiety Related Phenotypes in Mice. Humana Press, Totowa, NJ, pp 1–20

[CR23] Goulson D (2003) Bumblebees: Their behaviour and ecology. Oxford University Press

[CR24] Gumbert A (2000) Color choices by bumble bees (*Bombus terrestris*): innate preferences and generalization after learning. Behav Ecol Sociobiol 48:36–43

[CR25] Hagen M, Dupont YL (2013) Inter-tegular span and head width as estimators of fresh and dry body mass in bumblebees (*Bombus* spp.). Insectes Soc 60:251–257

[CR26] Hartig F (2025) DHARMa: Residual diagnostics for hierarchical (Multi-Level / Mixed) regression models

[CR27] Huth-Schwarz A, León A, Vandame R et al (2011) Workers dominate male production in the neotropical bumblebee *Bombus wilmattae* (Hymenoptera: Apidae). Front Zool 8:13 21651814 10.1186/1742-9994-8-13 PMC3127829

[CR28] Ings TC, Raine NE, Chittka L (2009) A population comparison of the strength and persistence of innate colour preference and learning speed in the bumblebee *Bombus terrestris*. Behav Ecol Sociobiol 63:1207–1218

[CR29] Izquierdo A, Jentsch JD (2012) Reversal learning as a measure of impulsive and compulsive behavior in addictions. Psychopharmacology 219:607–620 22134477 10.1007/s00213-011-2579-7 PMC3249486

[CR30] Kraus FB, Wolf S, Moritz RFA (2009) Male flight distance and population substructure in the bumblebee *Bombus terrestris*. J Anim Ecol 78:247–252 19120605 10.1111/j.1365-2656.2008.01479.x

[CR31] Lea SEG, Chow PKY, Leaver LA, McLaren IPL (2020a) Behavioral flexibility: a review, a model, and some exploratory tests. Learn Behav 48:173–187 32043268 10.3758/s13420-020-00421-w PMC7082303

[CR32] Lea SEG, Chow PKY, Leaver LA, McLaren IPL (2020b) Behavioral flexibility: a review, a model, and some exploratory tests. Learn Behav 48(1):173–187. 10.3758/s13420-020-00421-w 32043268 10.3758/s13420-020-00421-w PMC7082303

[CR33] Lenth R, Piaskowski J (2026) emmeans: Estimated marginal means, aka least-squares means. R package version 2.0.3 https://rvlenth.github.io/emmeans/

[CR34] Lucon-Xiccato T (2022) The contribution of executive functions to sex differences in animal cognition. Neurosci Biobehav Rev 138:104705. 10.1016/j.neubiorev.2022.104705 35605792 10.1016/j.neubiorev.2022.104705

[CR35] Lucon-Xiccato T, Bisazza A (2014) Discrimination reversal learning reveals greater female behavioural flexibility in guppies. Biol Lett 10:20140206

[CR36] Magnusson A, Skaug H, Nielsen A, et al (2017) glmmTMB: generalized linear mixed models using template model builder. R package version 0 1 3:

[CR37] Manning TH, Austin MW, MuseMorris K, Dunlap AS (2021) Equivalent learning, but unequal participation: male bumble bees learn comparably to females, but participate in cognitive assessments at lower rates. Behav Processes 193:104528 34626745 10.1016/j.beproc.2021.104528

[CR38] Montiglio P-O, Garant D, Thomas D, Réale D (2010) Individual variation in temporal activity patterns in open-field tests. Anim Behav 80:905–912

[CR39] Muth F, Tripodi AD, Bonilla R et al (2021) No sex differences in learning in wild bumblebees. Behav Ecol 32:638–645

[CR40] Ng VKY, Cribbie RA (2017) Using the gamma generalized linear model for modeling continuous, skewed and heteroscedastic outcomes in psychology. Curr Psychol 36:225–235

[CR41] Nicolakakis N, Sol D, Lefebvre L (2003) Behavioural flexibility predicts species richness in birds, but not extinction risk. Anim Behav 65:445–452

[CR42] Ogurtsov SV, Antipov VA, Permyakov MG (2018) Sex differences in exploratory behaviour of the common toad, *Bufo bufo*. Ethol Ecol Evol 30:543–568

[CR43] Osborne JL, Clark SJ, Morris RJ et al (1999) A landscape‐scale study of bumble bee foraging range and constancy, using harmonic radar. J Appl Ecol 36:519–533

[CR44] Pamminger T, Becker R, Himmelreich S et al (2019) The nectar report: quantitative review of nectar sugar concentrations offered by bee visited flowers in agricultural and non-agricultural landscapes. PeerJ 7:e6329 30834180 10.7717/peerj.6329 PMC6397631

[CR45] Raine NE, Chittka L (2008) The correlation of learning speed and natural foraging success in bumble-bees. Proc Biol Sci 275:803–808 18198141 10.1098/rspb.2007.1652 PMC2596909

[CR46] Raine NE, Chittka L (2012) No trade-off between learning speed and associative flexibility in bumblebees: a reversal learning test with multiple colonies. PLoS ONE 7:e45096 23028779 10.1371/journal.pone.0045096 PMC3447877

[CR47] Raine NE, Ings TC, Ramos-Rodriguez O, Chittka L (2006) Intercolony variation in learning performance of a wild British bumblebee population Hymenoptera: Apidae: *Bombus terrestris audax*. Entomol Gen 28:241

[CR48] Reader SM (2015) Causes of individual differences in animal exploration and search. Top Cogn Sci 7:451–468 25982255 10.1111/tops.12148

[CR49] Reader S M, Laland K N. (Eds.). (2003). *Animal innovation*. Oxford University Press.

[CR50] Réale D, Reader SM, Sol D et al (2007) Integrating animal temperament within ecology and evolution. Biol Rev Camb Philos Soc 82:291–318 17437562 10.1111/j.1469-185X.2007.00010.x

[CR51] Robert T, Frasnelli E, Collett TS, Hempel de Ibarra N (2017) Male bumblebees perform learning flights on leaving a flower but not when leaving their nest. J Exp Biol 220:930–937 27994042 10.1242/jeb.151126

[CR52] Rossi N, Doussot C, Woodgate JL, Lihoreau M, Chittka L (2025) Male bumblebees adapt foraging to environmental conditions to sustain mate-seeking efforts. bioRxiv. 10.1101/2025.09.08.67461541279868

[CR53] Rother L, Müller R, Kirschenmann E et al (2023) Walking bumblebees see faster. Proc Biol Sci 290:20230460 37192665 10.1098/rspb.2023.0460 PMC10188239

[CR54] Sokal RR, Rohlf FJ (1995) Biometry: the principles and practice of statistics in biological research. WH Freeman, New York New York, USA

[CR55] Strang CG, Sherry DF (2014) Serial reversal learning in bumblebees (*Bombus impatiens*). Anim Cogn 17:723–734 24218120 10.1007/s10071-013-0704-1

[CR56] Tanaka S, Young JW, Halberstadt AL, Masten VL, Geyer MA (2012) Four factors underlying mouse behavior in an open field. Behav Brain Res 233:55–61 22569582 10.1016/j.bbr.2012.04.045 PMC3866095

[CR57] Tapp PD, Siwak CT, Estrada J et al (2003) Size and reversal learning in the beagle dog as a measure of executive function and inhibitory control in aging. Learn Mem 10:64–73 12551965 10.1101/lm.54403 PMC196651

[CR58] Thomson JD, Plowright RC (1980) Pollen carryover, nectar rewards, and pollinator behavior with special reference to *Diervilla lonicera*. Oecologia 46:68–74 28310628 10.1007/BF00346968

[CR59] Toppa RH, Arena MVN, da Silva CI et al (2021) Impact of glues used for RFIDs on the longevity and flight muscles of the stingless bee Melipona quadrifasciata (Apidae: Meliponini). Apidologie 52:328–340

[CR60] Valterová I, Martinet B, Michez D et al (2019) Sexual attraction: a review of bumblebee male pheromones. Z Naturforsch C 74:233–250 31442206 10.1515/znc-2019-0003

[CR61] van Dixhoorn I, Aubé L, van Zyl C, de Mol R, van der Werf J, Lardy R, Mialon M-M, van Reenen K, Veissier I (2024) From data on gross activity to the characterization of animal behaviour: which metrics for which purposes? Peer Community Journal 4:e105. 10.24072/pcjournal.489

[CR62] Videlier M, Cornette R, Bonneaud C, Herrel A (2015) Sexual differences in exploration behavior in *Xenopus tropicalis*? J Exp Biol 218:1733–1739 25908061 10.1242/jeb.120618

[CR63] Walsh RN, Cummins RA (1976) The open-field test: a critical review. Psychol Bull 83:482–504 17582919

[CR64] Wascher CAF, Allen K, Szipl G (2021) Learning and motor inhibitory control in crows and domestic chickens. R Soc Open Sci 8:210504 34703616 10.1098/rsos.210504 PMC8527213

[CR65] Wolf S, Chittka L (2016) Male bumblebees, *Bombus terrestris*, perform equally well as workers in a serial colour-learning task. Anim Behav 111:147–155 26877542 10.1016/j.anbehav.2015.10.009 PMC4712640

[CR66] Wolf S, Toev T, Moritz RLV, Moritz RFA (2012) Spatial and temporal dynamics of the male effective population size in bumblebees (Hymenoptera: Apidae). Popul Ecol 54:115–124

[CR67] Yadav S, Yadav S, Sharma D, Sangwan N (2016) Bumblebees, Life Cycle and their role in pollination-A Review. Int J Res Sci.

[CR68] Zhao H, Liu Y, Zhang H et al (2021a) Worker-born males are smaller but have similar reproduction ability to queen-born males in bumblebees. Insects 12:1008 34821809 10.3390/insects12111008 PMC8622041

[CR69] Zhao H, Mashilingi SK, Liu Y, An J (2021b) Factors influencing the reproductive ability of male bees: current knowledge and further directions. Insects 12:529 34200253 10.3390/insects12060529 PMC8229853

