# Supplementary material for: Male bumblebees (Bombus terrestris) are more active and behaviourally flexible than workers
Source: Anim Cogn. 2026 Apr 10;29(1):41. doi: 10.1007/s10071-026-02061-5 (PMC13194259; doi:10.1007/s10071-026-02061-5)
Supplement: Supplementary file 1 [file 10071_2026_2061_MOESM1_ESM.docx]

Supplementary materials

**Table S1.** Activity task - Pairwise contrasts (Tukey correction on p values) of males’ and females’ frequency of visits to each compartment. This table shows estimates, standard error (S.E), *z* and *p* values. Bold values indicate significance, *p* <0.05.

**Table S2.** DLP between-group comparisons: 29 bees participated in the DLP task, and 10 males completed the DLP; 6 males did not complete the DLP, 11 females completed the DLP, and 2 females did not complete the DLP. Pairwise contrasts with Tukey adjusted *p* values were conducted to compare the four groups in A) inter-tengual span (ITS), B) sucrose consumption (ml), and C) total active time (minutes) in the activity task. Note that sample sizes vary due to two females in the completed group and one female in the incomplete group losing their tags before measuring body size. This table shows the contrast groups, estimates, standard errors (S.E), *Z* and *p* values. Bold values indicate *p* < 0.05. Also see figure 2.

**Note S1.** Individual analyses of bees’ learning performance

Table S1. activity task - Pairwise contrasts (Tukey correction on p values) of males’ and females’ frequency of visits to each compartment. This table shows estimates, standard error (S.E), *z* and *p* values. Bold values indicate significance, *p* <0.05

|  | Estimate | S. E | *z* | *p* |
| --- | --- | --- | --- | --- |
| Comp 1: Female - Male | -0.45 | 0.22 | -2.09 | **0.037** |
| Comp 2: Female - Male | -0.52 | 0.21 | -2.49 | **0.013** |
| Comp. 3: Female - Male | -0.69 | 0.22 | -3.20 | **0.001** |
| Comp. 4: Female - Male | -0.98 | 0.22 | -4.41 | **<0.001** |
| Comp. 5: Female - Male | -0.95 | 0.23 | -4.20 | **<0.001** |
| Comp. 6: Female - Male | -0.64 | 0.23 | -2.83 | **0.005** |
| Comp. 7: Female - Male | -0.40 | 0.23 | -1.77 | 0.077 |
| Comp. 8: Female - Male | -0.17 | 0.24 | -0.70 | 0.481 |
| Comp. 9: Female - Male | 0.13 | 0.25 | 0.52 | 0.604 |
| Comp. 10: Female - Male | 0.39 | 0.28 | 1.43 | 0.152 |

**Table S2.** DLP between-group comparisons: 29 bees participated in the DLP task, and 10 males completed the DLP; 6 males did not complete the DLP, 11 females completed the DLP, and 2 females did not complete the DLP. Pairwise contrasts with Tukey adjusted *p* values were conducted to compare the four groups in a) inter-tengual span (ITS), b) sucrose consumption (ml), and c) total active time (minutes) in the activity task. Note that sample sizes vary due to two females in the completed group and one female in the incomplete group losing their tags before measuring body size. This table shows the contrast groups, estimates, standard errors (S.E), *Z* and *p* values. Bold values indicate *p* < 0.05. Also see figure 2.

|  | Contrast | | Est | S.E | *Z* | *p* |
| --- | --- | --- | --- | --- | --- | --- |
| a) Inter-tegular span (ITS) | Female completed | Female not completed | -0.09 | 0.12 | -0.73 | 0.886 |
|  | Female completed | Male completed | -0.09 | 0.05 | -1.68 | 0.335 |
|  | Female completed | Male not completed | 0.10 | 0.06 | 1.67 | 0.342 |
|  | Female not completed | Male completed | <-0.01 | 0.12 | -0.01 | 1.000 |
|  | Female not completed | Male not completed | 0.19 | 0.12 | 1.52 | 0.424 |
|  | Male completed | Male not completed | 0.19 | 0.06 | 3.19 | **0.008** |
| b) Sucrose consumption (ml) | Female completed | Female not completed | 0.14 | 0.88 | 0.16 | 0.999 |
|  | Female completed | Male completed | 0.14 | 0.51 | 0.28 | 0.993 |
|  | Female completed | Male not completed | 5.44 | 0.59 | 9.27 | **<0.001** |
|  | Female not completed | Male completed | <-0.01 | 0.88 | <-0.01 | 1.000 |
|  | Female not completed | Male not completed | 5.30 | 0.93 | 5.71 | **<0.001** |
|  | Male completed | Male not completed | 5.30 | 0.59 | 9.04 | **<0.001** |
| c) Total active time (mins) | Female completed | Female not completed | 0.32 | 0.53 | 0.60 | 0.933 |
|  | Female completed | Male completed | -0.60 | 0.31 | -0.96 | 0.202 |
|  | Female completed | Male not completed | 0.29 | 0.35 | 0.82 | 0.847 |
|  | Female not completed | Male completed | -0.92 | 0.53 | -1.73 | 0.309 |
|  | Female not completed | Male not completed | -0.03 | 0.56 | -0.05 | 1.000 |
|  | Male completed | Male not completed | 0.89 | 0.35 | 2.52 | 0.058 |

Note S1. *Individual analyses of bees’ performance in early RLP*

15 bees (8 females and 7 males) participated in the RLP. To understand whether bees had learned the DLP colour-reward association (and not the simply used hypothetical direct cues from the sugar solution), we conducted individual analyses. To do so, we examined the consecutive incorrect choices (previously rewarded colour in the DLP but now unrewarded in the RLP) that each bee made in their first 5 choices of the first RLP session. The use of 5 choices as the cut-off point was based on consecutive probability in reaching significance (p = 0.031) as well as accounting for the possibility that bees may start learning the new reward contingency toward the end of the sessions. We pooled the *P* values of individuals within the same-sex group using Fisher's formula *χ*2 = −2 Σ*In*(*P*) [(Sokal and Rohlf 1995)](https://paperpile.com/c/2smMMw/nYaWU). Results showed that both sexes chose the previously rewarded colour significantly more than at random (pooled consecutive binomial tests: females: *χ*2_16_ = 41.59, p < 0.001; Males: *χ*2_14_ = 30.50, p < 0.01), and therefore had learned the previous colour-reward contingency. It also suggests that they did not rely on other cues (e.g., distinguishing the small sugar solution drop on the rewarded flower from the water drop on the unrewarded ones to make their choice) as the choices would likely be biased in the opposite direction (already more correct than at random early in the RLP).
